# Book Notices

**Published:** 1885-06

**Authors:** 


### BOOK NOTICES.

THE DISEASES OF THE EAR, AND THEIR HOMŒOPATHIC TREATMENT, WITH A BRIEF OUTLINE OF THEIR ANATOMY, PHYSIOLOGY, AND PATHOLOGY. By Charles Frederick Sterling, M. D., O. Ch. A. Chir. Pages 167; price, $1.25. New York: A. L. Chatterton Publishing Company, 1885.

The author presents this little work on the diseases of the ear "as a manual for the student and general practitioner." A small, concise manual on these diseases is doubtless very much needed by the general practitioner, and will fill a "general want." On looking over Dr. Sterling's manual, we find the anatomy and the diseases of the ear are clearly and concisely treated. Indications for the remedies advised are, in the main, well stated. In one or two places we find objectionable local measures advised. We do not believe Majendie's or any other opiate is necessary to relieve the pain—homœopathic therapeutics can easily excel any opiate as a "pain killer." Nor can we acknowledge our therapeutics to be so impotent as to justify this statement: "Local measures are indispensable. One might as soon attempt to treat a sloughing ulcer by medicine alone (!), as to think most of these cases (*i. e.*, suppuration of middle ear) can be cured by a few powders or pellets." With these exceptions, we think Dr. Sterling's book a good one.

DISEASES OF THE NARES, LARYNX, AND TRACHEA IN CHILDHOOD. By Thomas Nichol, M. D., LL. D., S. C. L. Pages 302; price, $2.50. New York: A. L. Chatterton Publishing Company, 1885.

Those diseases which are most frequently met, and which are most intractable or dangerous, are those that this book considers. Coryza, acute and chronic, laryngitis, croup, etc., are about the worst troubles which threaten children. It is, therefore, no useless book which Dr. Nichol presents the practitioner. Dr. Nichol gives very full consideration to the pathology, diagnosis, etc., of these diseases; his treatment of them is also very good in most cases. Only he seems to have great respect for the authority, the *ipse dixit*, of authors and writers. All through the book we find remedies quoted seemingly because recommended by Dr. So-and-so! This appears very curious to a homœopathist; we always thought our remedies were prescribed according to *law*, and a remedy given for the symptoms of the patient, not because A or B recommended it as good for *a disease*. A remedy may be called for by the symptoms, although never before, to the prescriber's knowledge, used, in a case of croup, for instance. We feel sure Dr. Nichol knows and appreciates all this as well as any one, yet his book seems to us to convey a wrong impression to his readers.

The "aphorisms" given at the end of the chapters contain some very good hints as to causes and prevention of these diseases. Dr. Nichol has evidently devoted much time and study to this work, and has produced a work whose value is much above the average of our homœopathic publications. It is to be hoped we shall soon have his promised work on the diseases of the lungs and bronchi. We would like to add one "aphorism;" it is this: Prescribe for the patient, *not for the disease*. Do not be afraid to use the higher dilutions; and, above all, do not be frightened because the disease you are treating has a *bad name* (given to it by allopathic incompetency). Your potencies will conquer *that* if carefully selected.

